# Supplementary material for: Leishmania sand fly-transmission is disrupted by Delftia tsuruhatensis TC1 bacteria
Source: Nat Commun. 2025 May 8;16:3571. doi: 10.1038/s41467-025-58769-4 (PMC12062286; doi:10.1038/s41467-025-58769-4)
Supplement: Supplementary file 1 — Supplementary Information [file 41467_2025_58769_MOESM1_ESM.pdf]

Supplementary Information for

Leishmania sand fly-transmission is disrupted by *Delftia tsuruhatensis* TC1 bacteria

Pedro Cecilio[1]*, Luana A. Rogerio[2], Tiago D. Serafim[2], Kristina Tang[2], Laura Willen[2], Eva Iniguez[2], Claudio Meneses[2], Luis F. Chaves[3], Yue Zhang[4], Luiza dos Santos Felix[2], Wei Huang[5], Melina Garcia Guizzo[1], Pablo Castañeda-Casado[6], Marcelo Jacobs-Lorena[5], Jesus G. Valenzuela[2], Janneth Rodrigues[6]*, Fabiano Oliveira[2]*

Corresponding authors: Pedro Cecilio (pedro.amadocecilio@nih.gov), Janneth Rodrigues (janneth-fatima-indira.x.rodrigues@gsk.com), and Fabiano Oliveira (loliveira@niaid.nih.gov).

The PDF file includes:

Figs. S1 to S5

Table S1

Table S2

Data S1

Data S2

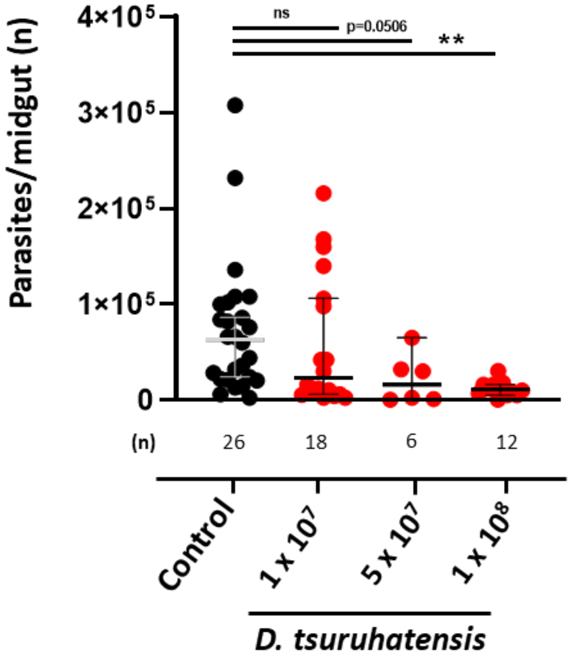

Fig. S1. Effect of the dose of *Delftia tsuruhatensis* on the *Leishmania* infection phenotype in sand flies. Sand flies were allowed to take a sugar meal (5% sterile sucrose solution), alone (control), or containing different concentrations of *Delftia tsuruhatensis* (numbers in the x axis/ml), and then infected with *L. major* parasites via artificial membrane feeding. Midguts were dissected at 11 days post-bloodmeal and the infection burden was quantified microscopically. The total number of parasites per midgut of control (black dots) and bacteria-fed (red dots) sand flies is represented. Each dot represents a single midgut. The group median and 95% CI are also shown. Statistical significance was determined using the Kruskal-Wallis test followed by a post-hoc analysis with the Dunn's test. The results of the post-hoc analysis are highlighted in the graph: ns – not significant; and **p=0.0015. Of note one exact p value is shown due to its "borderline significance". All results were obtained in, at least, two independent experiments.

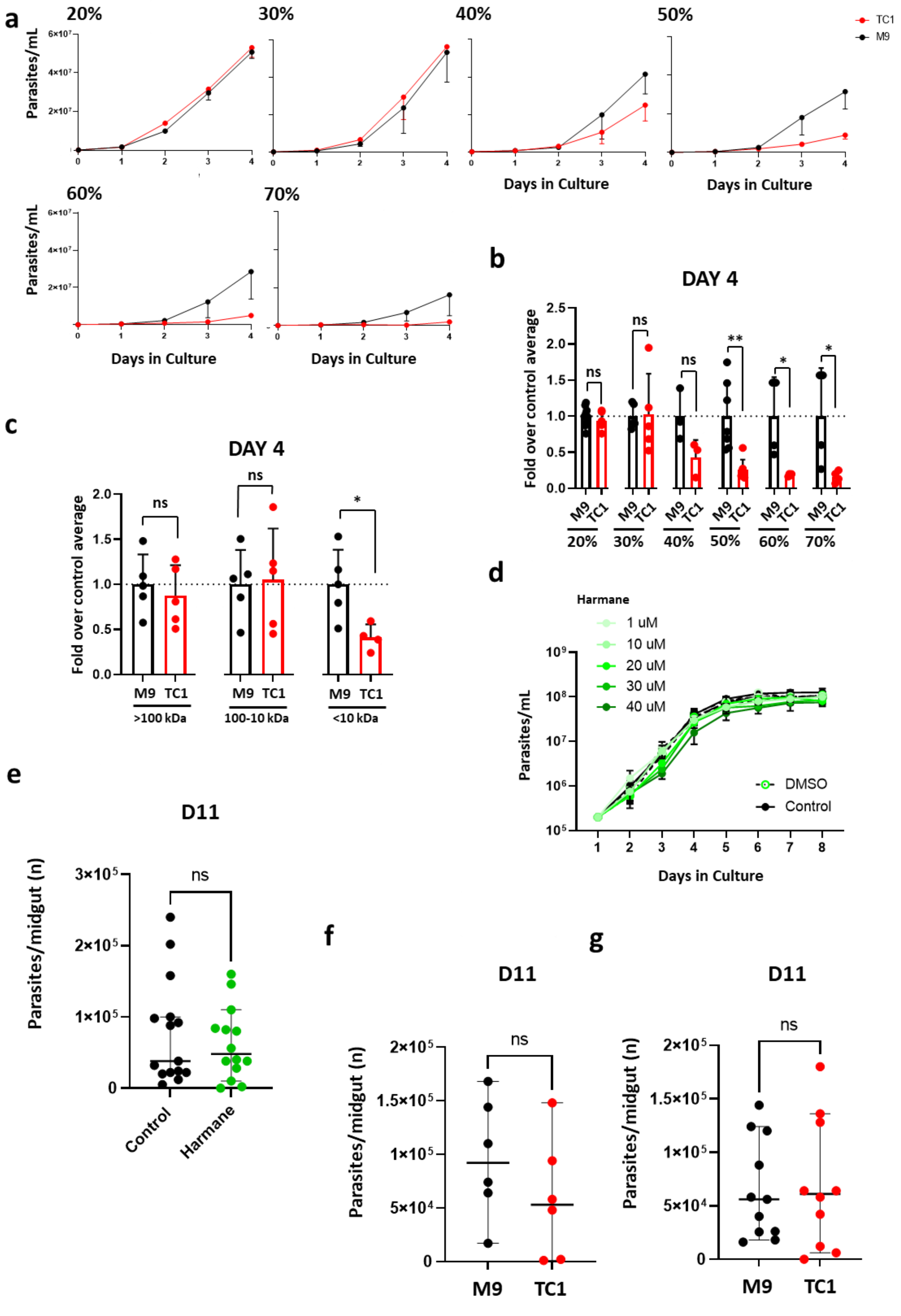
a
20%
30%
40%
50%
60%
70%
TC1
M9
Parasites/mL
Days in Culture
b
DAY 4
Fold over control average
ns
**
*
M9
TC1
20%
30%
40%
50%
60%
70%
c
DAY 4
>100 kDa
100-10 kDa
<10 kDa
d
Harmane
1 uM
10 uM
20 uM
30 uM
40 uM
DMSO
Control
e
D11
Parasites/midgut (n)
Control
Harmane
f
D11
g
D11

Fig. S2. Although *Delftia tsuruhatensis* excreted/secreted products other than harmane impact the growth of *Leishmania* parasites *in vitro*, the phenotype is not observed *in vivo* within the sand fly gut. (**a-b**) Increasing amounts of bacteria-free culture supernatant (in M9 medium – named as TC1 in the figure) were added to $1x10^5$ *Leishmania* parasites, and growth was monitored daily. Control wells received M9 medium subjected to the same conditions in the absence of bacteria (named M9 in the figure). (**a**) The growth curves obtained for parasites treated with bacteria-free TC1 (red lines) or control M9 (black lines) culture supernatant are represented. Median values with 95% CI are plotted. (**b**) The parasite numbers determined at day 4 of culture were normalized over the average of the respective control group for all supernatant concentrations tested. Normalized values for parasites treated with bacteria-free TC1 (red dots) or control M9 (black dots) culture supernatant are represented, together with the mean and SD values. Each dot represents a single well. (**c**) The same *in vitro* experimental approach described for panels **a**-**b** was used in the frame of amicon-fractionated supernatant pools: > 100 kDa, 100-10 kDa, and <10 kDa. Data are represented as in **b**. (**d**) The same *in vitro* experimental approach described for panels **a**-**b** was used in the frame of harmane treatment. Increasing concentrations of harmane were added to *Leishmania* parasites; control parasites were either treated with DMSO, or not treated at all. The growth curves obtained for harmane-treated (different shades of green dots and lines), DMSO-control (opened light green circles connected with black dotted lines), or untreated-control (black dots and lines) parasites are represented. Median values with 95% CI are plotted. (**e**) Sand flies were infected with *L. major* parasites via artificial membrane feeding in the presence or absence of 50 μM. harmane, and then maintained with sugar *ad libitum*. The total number of parasites per midgut of control (black dots) and harmane-fed (green dots) sand flies, 11 days post-infection is represented. Each dot represents a single midgut. The

group median and 95% CI are also shown. (**f**) Sand flies were infected with *L. major* parasites via artificial membrane feeding in the presence or absence of 50% bacteria-free culture supernatant, and then maintained with sugar *ad libitum*. The total number of parasites per midgut of control supernatant- (black dots) and bacteria-free supernatant- (red dots) fed sand flies, 11 days post-infection is represented. Each dot represents a single midgut. The group median and 95% CI are also shown. (**g**) Sand flies were infected with *L. major* parasites via artificial membrane feeding and separated into 2 groups. One group received 100% bacteria-free TC1 supernatant as the sugar meal vehicle daily, while the control group received M9 control supernatant in the same context. The total number of parasites per midgut of control M9 supernatant- (black dots) and bacteria-free TC1 supernatant- (red dots) fed sand flies, 11 days post-infection is represented. Each dot represents a single midgut. The group median and 95% CI are also shown. Statistical significance was determined using the Mann-Whitney test or Unpaired t-test (both two-tailed; for non-parametric, and parametric data, respectively; more details available in the source file) and is highlighted: (**b**, **c**, **e**-**g**) ns – not significant (p>0.05); (**b**) *p=0.0237 or *p=0.0473 (for the 60 and 70% comparisons, respectively), and **p=0.0017; (**c**) *p=0.0240. All results were obtained in, at least, two independent experiments.

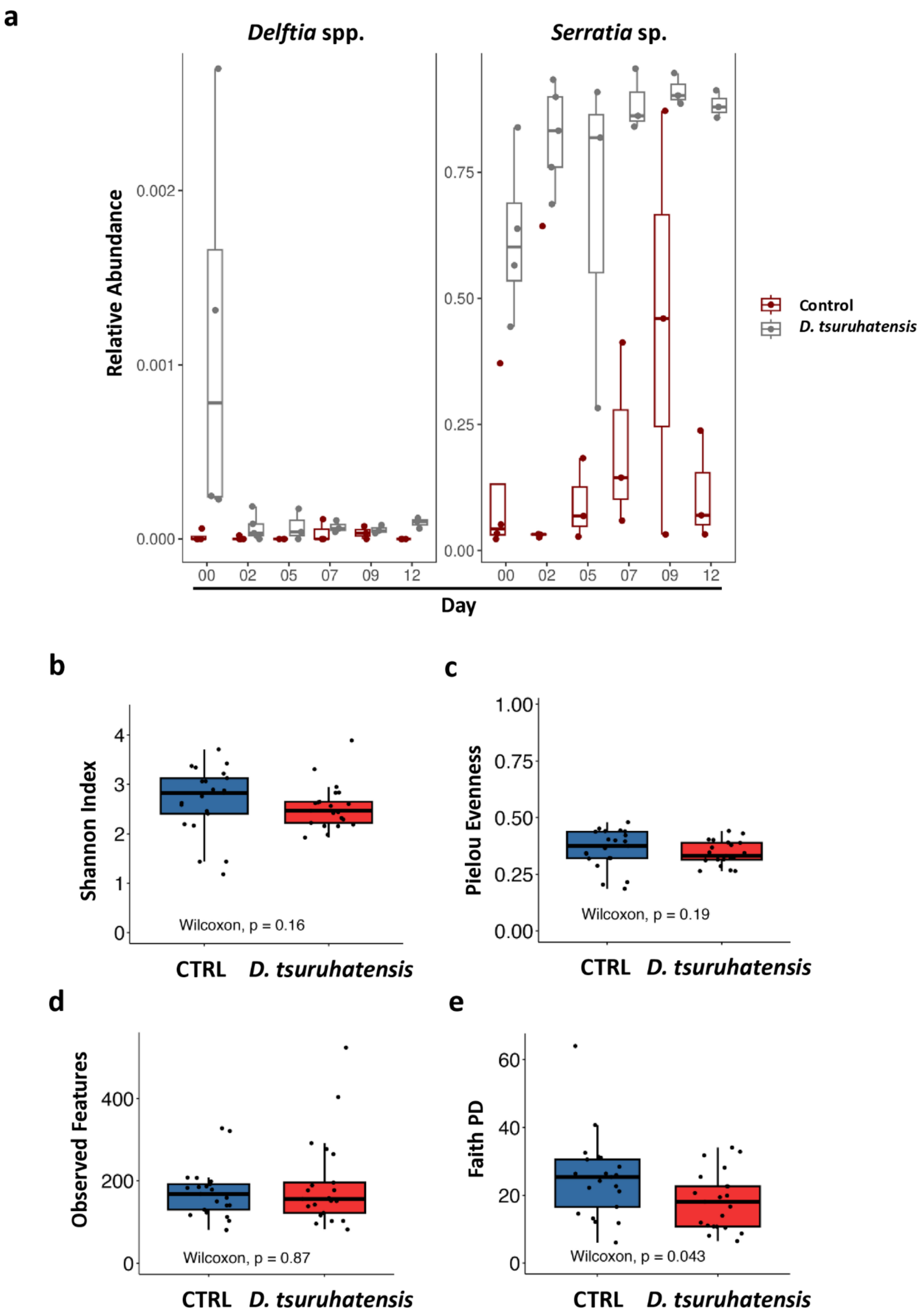
a
Delftia spp.
Serratia sp.
Relative Abundance
0.000
0.001
0.002
0.00
0.25
0.50
0.75
00
02
05
07
09
12
Day
Control
D. tsuruhatensis
b
Shannon Index
Wilcoxon, p = 0.16
CTRL
D. tsuruhatensis
c
Pielou Evenness
Wilcoxon, p = 0.19
CTRL
D. tsuruhatensis
d
Observed Features
Wilcoxon, p = 0.87
CTRL
D. tsuruhatensis
e
Faith PD
Wilcoxon, p = 0.043
CTRL
D. tsuruhatensis

Fig. S3. The relative abundance of *Serratia* spp. is much higher than that of *Delftia* spp. (particularly in the midguts of *Delftia tsuruhatensis*-treated sand flies), but the bacterial species alpha diversity is not significantly altered in the midguts of control versus *Delftia tsuruhatensis*-treated sand flies. Sand flies were allowed to take a sugar meal (5% sterile sucrose solution), alone (control), or containing *D. tsuruhatensis* ($1x10^8$/ml) and then infected with *L. major* parasites via artificial membrane feeding. Pools of sand fly midguts were collected one day after bacterial (or control sugar) feeding and prior to infection (day 0), as well as 2, 5, 7, 9, and 12 days after infection and subjected to metagenomics analysis. (**a**) Relative abundance of *Delftia* spp. (left panel) and *Serratia* sp. (right panel) in *Delftia tsuruhatensis*-treated (grey box-and-whiskers) and control (bordeaux box-and-whiskers) sand flies, per time-point. (**b-d**) Four different indicators were used to evaluate the alpha diversity at the group level. Box-and-whisker graphs show an overview of the calculated Shannon Index (**b**), Pielou Evenness (**c**), Observed Features (**d**), and Faith PD (**e**) values per pool of *Delftia tsuruhatensis*-treated (red; n=21) and control (blue; n=21) sand fly midgut pools collected. Statistical significance was determined and is highlighted.

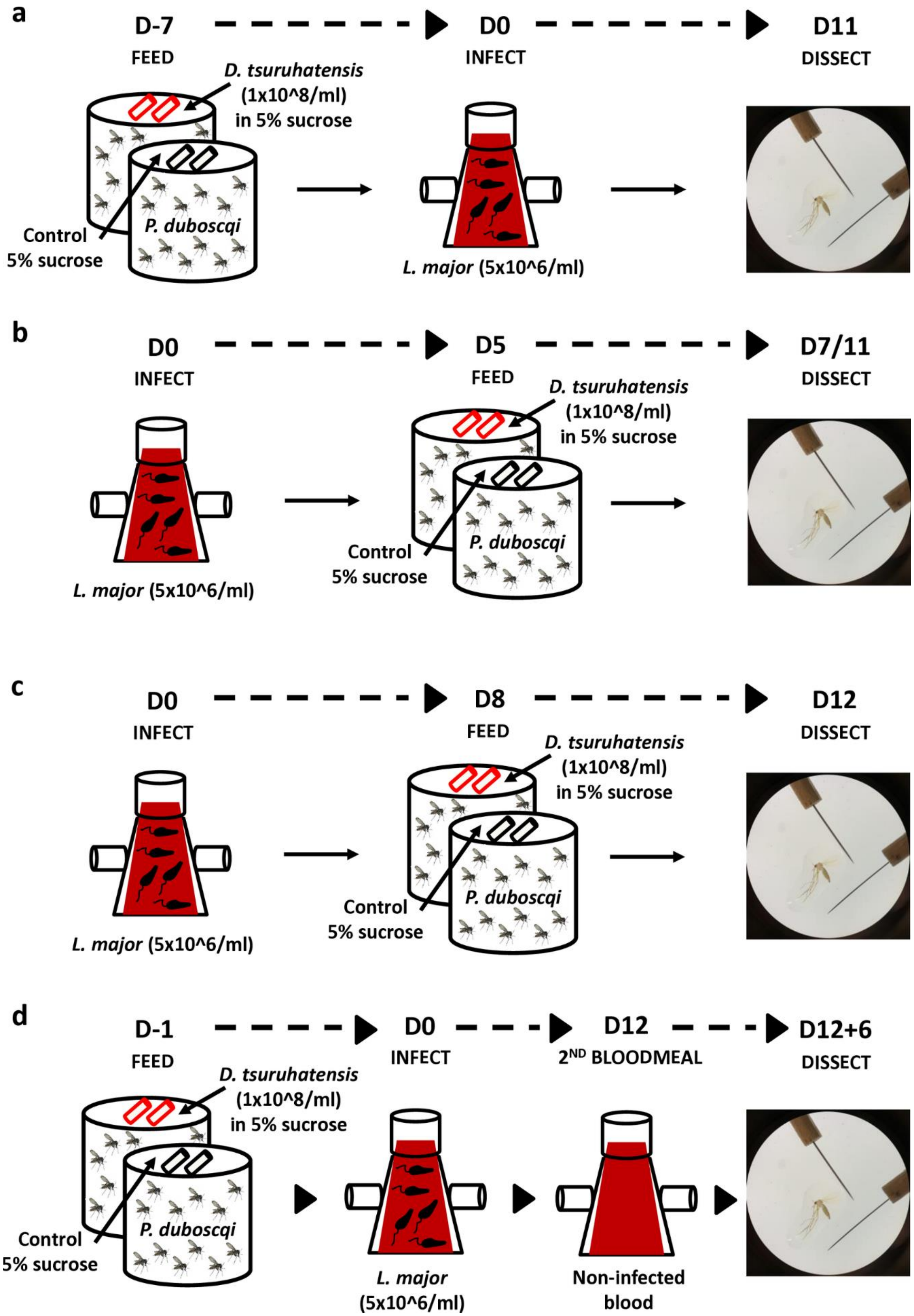

a
D-7
FEED
D. tsuruhatensis
(1x10^8/ml)
in 5% sucrose
Control
5% sucrose
P. duboscqi
D0
INFECT
L. major (5x10^6/ml)
D11
DISSECT
b
D0
INFECT
L. major (5x10^6/ml)
D5
FEED
D. tsuruhatensis
(1x10^8/ml)
in 5% sucrose
Control
5% sucrose
P. duboscqi
D7/11
DISSECT
c
D0
INFECT
L. major (5x10^6/ml)
D8
FEED
D. tsuruhatensis
(1x10^8/ml)
in 5% sucrose
Control
5% sucrose
P. duboscqi
D12
DISSECT
d
D-1
FEED
D. tsuruhatensis
(1x10^8/ml)
in 5% sucrose
Control
5% sucrose
P. duboscqi
D0
INFECT
L. major
(5x10^6/ml)
D12
2ND BLOODMEAL
Non-infected
blood
D12+6
DISSECT

Fig. S4. Representation of the experimental layouts used to determine whether the *Delftia tsuruhatensis* impact on the development of *Leishmania* parasites within the sand fly midgut is time-dependent. (**a**) Experimental layout used to obtain the data reported in Fig. 4a. (**b**) Experimental layout used to obtain the data reported in Fig. 4b. (**c**) Experimental layout used to obtain the data reported in Fig. 4c. (**d**) Experimental layout used to obtain the data reported in Fig. 4d.

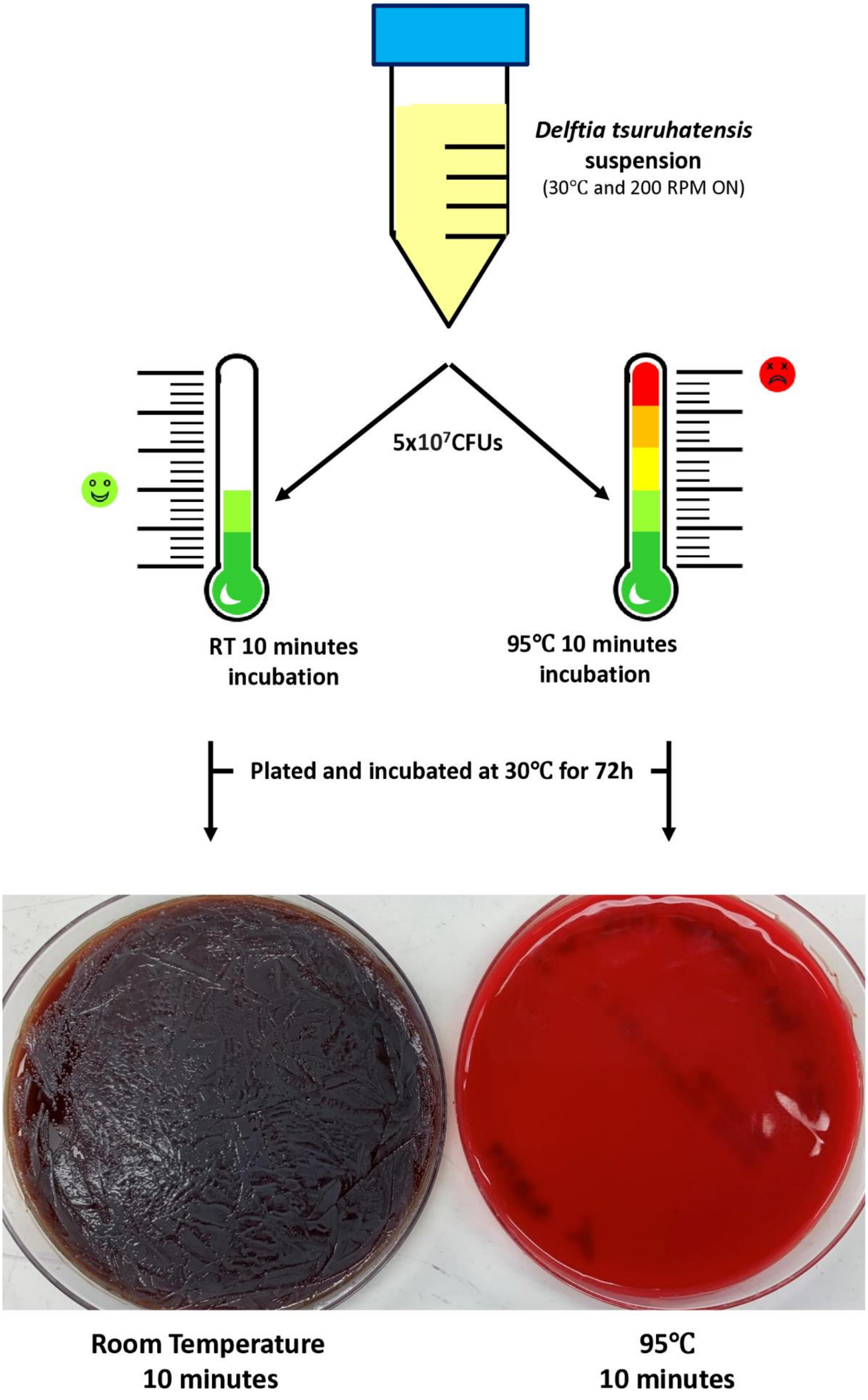

Delftia tsuruhatensis
suspension
(30°C and 200 RPM ON)
$5x10^7$CFUs
RT 10 minutes
incubation
95°C 10 minutes
incubation
Plated and incubated at 30°C for 72h
Room Temperature
10 minutes
95°C
10 minutes

Figure S5. Heat-inactivation kills *Delftia tsuruhatensis* bacteria. *D. tsuruhatensis* TC1 were grown overnight in LB broth at 30 °C under agitation (200 RPM) and washed with sterile PBS via sequential centrifugation steps (3500 RPM, 10 minutes 4 °C). Two aliquots of $5x10^7$ colony forming units (CFUs) were then suspended in 70 µl of sterile PBS. One aliquot was then kept in the lab bench (control), while the other was incubated at 95 °C for 10 minutes. Soon after, each suspension was plated via spreading into an individual blood agar plate, and plates were incubated at 30 °C for 3 days. Images of both plates after the incubation period are shown.

Table S1. Absolute abundance of *Delftia* spp. and *Serratia* sp. in the sand fly midgut: ANCOM-BC analysis output, including statistics.

Day	Genus	*D.tsuruhatensis*-fed versus Control		p value
		Log(Fold Change)	Standard Error	
0	*Delftia* spp.	4.052630955	0.737807231	3.96E-08
0	*Serratia* sp.	2.235875579	0.501748404	8.34E-06
2	*Delftia* spp.	1.545473369	0.605323733	0.010676
2	*Serratia* sp.	2.674486401	0.550905086	1.21E-06
5	*Delftia* spp.	1.755314533	0.929287116	0.058908
5	*Serratia* sp.	2.115648797	0.493376165	1.80E-05
7	*Delftia* spp.	1.479420119	0.800010218	0.064422
7	*Serratia* sp.	1.744274487	0.508465065	0.000603
9	*Delftia* spp.	0.641872254	0.687295827	0.35035
9	*Serratia* sp.	1.313312164	0.871218593	0.131697
12	*Delftia* spp.	2.712457358	0.169897668	2.23E-57
12	*Serratia* sp.	2.394577374	0.492855713	1.18E-06

Table S2. Parameters employed to estimate changes in R_0 following the colonization of the sand fly midgut with *Delftia tsuruhatensis* TC1

**Parameter**	**Estimate ± SD**
R_0 cross sectional study in dogs[1]	1.20 ± 0.09
R_0 cross sectional study multiple hosts[2]	1.271 ± NA
R_0 time series of human cases[3]	1.90 ± NA
Daily average survival – control*	0.020 ± 0.014
Daily average survival – treatment*	0.032 ± 0.019
Infection rate – control	0.51 ± 0.08
Infection rate – treatment	0.33 ± 0.05

NA = Not Reported, * 14-days mortality estimates are obtained by multiplying these daily estimates times 14 (see code S1 for details). Daily mortality estimates are based on the natural logarithm[4] of the average daily survival from the different experiments.

Data S1. Differences in bacterial abundance in the gut of control *versus D. tsuruhatensis*-fed sand flies. This table is available as a separate .xlsx file.

Data S2. R code used for the modeling approach. This code is available as a separate .docx file.

References

1 Calzada, J. E. *et al.* Cutaneous Leishmaniasis in dogs: is high seroprevalence indicative of a reservoir role? *Parasitology* **142**, 1202-1214, doi:10.1017/S0031182015000475 (2015).

2 Chaves, L. F., Hernandez, M. J. & Ramos, S. Simulacion de modelos matematicos como herramienta para el estudio de los reservorios de la Leishmaniasis Cutanea Americana. *Divulgaciones Matematicas* **16**, 125-154 (2008).

3 Chaves, L. F. Climate and recruitment limitation of hosts: the dynamics of American cutaneous leishmaniasis seen through semi-mechanistic seasonal models. *Annals of Tropical Medicine & Parasitology* **103**, 221-234, doi:10.1179/136485909X398267 (2009).

4 Carey, J. R. Insect biodemography. *Annu Rev Entomol* **46**, 79-110, doi:10.1146/annurev.ento.46.1.79 (2001).
