# Supplementary material for: Leishmania sand fly-transmission is disrupted by Delftia tsuruhatensis TC1 bacteria
Source: Nat Commun. 2025 May 8;16:3571. doi: 10.1038/s41467-025-58769-4 (PMC12062286; doi:10.1038/s41467-025-58769-4)
Supplement: Supplementary file 3 — Description of Additional Supplementary Files [file 41467_2025_58769_MOESM3_ESM.pdf]

Description of Additional Supplementary Files

Data S1. Differences in bacterial abundance in the gut of control *versus D. tsuruhatensis*-fed sand flies.

Data S2. R code used for the modeling approach.
