# Supplementary material for: Leishmania sand fly-transmission is disrupted by Delftia tsuruhatensis TC1 bacteria
Source: Nat Commun. 2025 May 8;16:3571. doi: 10.1038/s41467-025-58769-4 (PMC12062286; doi:10.1038/s41467-025-58769-4)
Supplement: Supplementary file 5 — Supplementary Data 2 [file 41467_2025_58769_MOESM5_ESM.pdf]

Data S2. R code used for the modeling approach

```
#Code to generate R_0 surfaces evaluating potential impacts of sand fly infection
#Assuming BC/gamma to be constant, we make R_0 a function of just beta and mu
#We can generate a surface that is a function of beta^2 and mu
#We start by generating a range of values for parameters mu and beta

mu<-50:750/1000
beta2<-2*(300:550)^2/1000000


## Here we generate a R_0 surface

r0<-outer(beta2,1/mu)


#We further load a couple of libraries that are useful to generate color palettes

library(RColorBrewer)
library("viridis")


### In the first panel, we only study the impact of sand fly mortality
### Infected sand flies die, on average 1.6 times more when exposed to the bacteria
### R0 estimates are 1.20 in dogs, 1.271 in a multi-host system with donkeys as reservoirs,
### and 1.90 from a human case time series. Refer to Table S1 for further details.

### We estimated the mortality based on data from our experiments
### The mortality for the control group is an estimate based on
### pooling survival (l_X) estimates from the experiments where survival of sand flies
### was compared when exposed to the bacteria, the bacteria plus blood, and the bacteria plus infected blood.
### The control estimate was based on the following survival schedule (based on daily observations):

l_x_control=c(100,
        98.9862069,
        97.51034483,
        92.90689655,
        90.33655172,
        88.66758621,
        87.17965517,
        85.53103448,
        85.41896552,
        82.63413793,
        82.01344828
)


### With this, it is possible to calculate the probability of death:

p_x_control= l_x_control[-1]/l_x_control[-length(l_x_control)]


### which can be used to obtain daily mortality,
```

assuming mortality is described by an exponential model (i.e., constant mortality):

mu_x_control=mean(-log(p_x_control))

We can then estimate mortality for a period of 14 days,
by multiplying 14 times the estimate for daily probability of death:

mu_x_c_14 = log((exp(mu_x_control))^14)

We also estimated the mortality for sand flies that were exposed to the bacteria:

```
l_x_bacteria = c(100,
        97.06322581,
        94.65354839,
        88.49129032,
        84.74967742,
        82.66612903,
        81.29032258,
        79.47354839,
        79.19129032,
        74.83290323,
        72.94290323
)

p_x_bacteria= l_x_bacteria[-1]/l_x_bacteria[-length(l_x_bacteria)]
mu_x_bacteria=mean(-log(p_x_bacteria))
mu_x_b_14 = log((exp(mu_x_bacteria))^14)

pal <- c(brewer.pal(7,"Oranges"))
tiff(filename = "panelmortality.tif",
   width = 10, height = 8, units = "cm", pointsize = 6,
   compression = "lzw",res=1200)
par(mar=c(5,5,.35,.35))
cuts=c(0,.5,1,1.5,2,5,10,20) #set breaks
image(beta2,mu,(r0),col =
pal,breaks=cuts,ylab=expression((mu)),xlab=expression(beta^2),cex.lab=1.5,cex.axis=1.5)
contour(beta2,mu,(r0),add=TRUE,lwd=1.2,levels=c(.5,1,1.5,2,5,10),labcex=1)
```

We can then plot in the surface R0 values,
assuming an average 14-day mortality in control sand flies of 0.278,
and an average mortality in bacteria-treated sand flies of 0.441, getting the coordinates

```
dogs1<-c(1.20*(mu_x_c_14),(mu_x_c_14))
dogs2<-c(1.20*(mu_x_c_14),(mu_x_b_14))


arrows(x0=1.20*(mu_x_c_14),y0=(mu_x_c_14),x1=1.20*(mu_x_c_14),y1=(mu_x_b_14),lwd=2,col="white",le
ngth = 0.075)
arrows(x0=1.271*(mu_x_c_14),y0=(mu_x_c_14),x1=1.271*(mu_x_c_14),y1=(mu_x_b_14),lwd=2,col="white",
length = 0.075)
arrows(x0=1.90*(mu_x_c_14),y0=(mu_x_c_14),x1=1.90*(mu_x_c_14),y1=(mu_x_b_14),lwd=2,col="white",le
ngth = 0.075)
text(0.34,0.25,"Dogs",cex=1.25)
```

```
text(0.398,0.33,"Multiple Hosts",cex=1.25)
text(0.555,0.31,"Humans",cex=1.25)
points(c((mu_x_c_14),(mu_x_c_14),(mu_x_c_14))~c(1.20*(mu_x_c_14),1.271*(mu_x_c_14),1.90*(mu_x_c_14)
),pch=15,col="white")
points(c((mu_x_c_14),(mu_x_c_14),(mu_x_c_14))~c(1.20*(mu_x_c_14),1.271*(mu_x_c_14),1.90*(mu_x_c_14)
),pch=1,cex=0.75)
dev.off()


### In the second panel we add the investigation of the potential impact of the bacteria on transmission
### We estimate a change in parameter beta from the R_0 equation.
### For this we combine results from our experiments looking at parasite pickup,
### and the development of metacyclic forms in the midgut.
### We specifically estimated the quality of our infections in the group of sand flies exposed to the bacteria.
### From our results, in the control group 22 out 43 sand flies had metacyclic parasites in the midgut
### and in the bacteria-treated group 13 out of 42 sand flies had metacyclic parasites in the midgut.
### We can then assume that beta^2 is modified by the resulting ratio
### of disseminating infections in the bacteria treatment divided by the control treatment.
### This ratio, beta_change, is then the following:

beta_change=(13/42)/(22/43)

tiff(filename = "panelmortalitybiting.tif",
    width = 10, height = 8, units = "cm", pointsize = 6,
    compression = "lzw",res=1200)
par(mar=c(5,5,.35,.35))

image(beta2,mu,(r0),col =
pal,breaks=cuts,ylab=expression((mu)),xlab=expression(beta^2),cex.lab=1.5,cex.axis=1.5)
contour(beta2,mu,(r0),add=TRUE,lwd=1.2,levels=c(.5,1,1.5,2,5,10),labcex=1)
text(0.34,0.25,"Dogs",cex=1.25)
text(0.388,0.30,"Multiple Hosts",cex=1.25)
text(0.551,0.30,"Humans",cex=1.25)

arrows(x0=1.20*(mu_x_c_14),y0=(mu_x_c_14),x1=1.20*(mu_x_c_14)*beta_change,y1=(mu_x_b_14),lwd=2,c
ol="white",length = 0.075)
arrows(x0=1.271*(mu_x_c_14),y0=(mu_x_c_14),x1=1.271*(mu_x_c_14)*beta_change,y1=(mu_x_b_14),lwd=2
,col="white",length = 0.075)
arrows(x0=1.90*(mu_x_c_14),y0=(mu_x_c_14),x1=1.90*(mu_x_c_14)*beta_change,y1=(mu_x_b_14),lwd=2,c
ol="white",length = 0.075)
points(c((mu_x_c_14),(mu_x_c_14),(mu_x_c_14))~c(1.20*(mu_x_c_14),1.271*(mu_x_c_14),1.90*(mu_x_c_14)
),pch=15,col="white")
points(c((mu_x_c_14),(mu_x_c_14),(mu_x_c_14))~c(1.20*(mu_x_c_14),1.271*(mu_x_c_14),1.90*(mu_x_c_14)
),pch=1,cex=1)
dev.off()
```
