# Supplementary material for: Leishmania sand fly-transmission is disrupted by Delftia tsuruhatensis TC1 bacteria
Source: Nat Commun. 2025 May 8;16:3571. doi: 10.1038/s41467-025-58769-4 (PMC12062286; doi:10.1038/s41467-025-58769-4)
Supplement: Supplementary file 6 — Reporting Summary [file 41467_2025_58769_MOESM6_ESM.pdf]

nature portfolio

Corresponding author(s): Pedro Cecilio
Janneth Rodrigues
Fabiano Oliveira

Last updated by author(s): 3/25/2025

Reporting Summary

Nature Portfolio wishes to improve the reproducibility of the work that we publish. This form provides structure for consistency and transparency in reporting. For further information on Nature Portfolio policies, see our Editorial Policies and the Editorial Policy Checklist.

Statistics

For all statistical analyses, confirm that the following items are present in the figure legend, table legend, main text, or Methods section.

n/a	Confirmed	
☐	☒	The exact sample size (*n*) for each experimental group/condition, given as a discrete number and unit of measurement
☐	☒	A statement on whether measurements were taken from distinct samples or whether the same sample was measured repeatedly
☐	☒	The statistical test(s) used AND whether they are one- or two-sided<br>*Only common tests should be described solely by name; describe more complex techniques in the Methods section.*
☐	☒	A description of all covariates tested
☐	☒	A description of any assumptions or corrections, such as tests of normality and adjustment for multiple comparisons
☐	☒	A full description of the statistical parameters including central tendency (e.g. means) or other basic estimates (e.g. regression coefficient) AND variation (e.g. standard deviation) or associated estimates of uncertainty (e.g. confidence intervals)
☐	☒	For null hypothesis testing, the test statistic (e.g. *F*, *t*, *r*) with confidence intervals, effect sizes, degrees of freedom and *P* value noted<br>*Give P values as exact values whenever suitable.*
☒	☐	For Bayesian analysis, information on the choice of priors and Markov chain Monte Carlo settings
☒	☐	For hierarchical and complex designs, identification of the appropriate level for tests and full reporting of outcomes
☒	☐	Estimates of effect sizes (e.g. Cohen's *d*, Pearson's *r*), indicating how they were calculated

Our web collection on statistics for biologists contains articles on many of the points above.

Software and code

Policy information about availability of computer code

Data collection	Illumina NovaSeq Control v1.7.5; Leica Application Suite X v3.7.4
Data analysis	GraphPad Prism v9; Imaris v9.2.1; QIIME2 2021.4; SPAdes 3.15.5 (for metagenome assembly); GTDB-Tk 2.1.1; metaWRAP 1.2.1; R v4.2.3; RStudio 2022.07.1+554. metaWRAP dependencies: general – python 2.7, shell; read_qc – fastqc v0.11.8, trim_galore 0.5.0, bmtagger 3.101, human_db hg38; binning – bwa 0.7.17, samtools 1.9, metabat2 2.12.1, concoct 1.0.0, MaxBin2 2.2.6; bin_refinement – bwa 0.7.17, samtools 1.9, kraken 1.1.1, kraken_db STANDARD_DATABASE, kronatools 2.7.1, checkm v1.0.12, checkm_DB (standard), SPAdes v3.13.0; quant_bins – salmon v0.13.1, seaborn 0.9.0. R packages: iCAMP_1.5.12, jakR_0.11.9, RColorBrewer_1.1-3, wesanderson_0.3.6, rstatix_0.7.2, ggsci_3.0.0, metagMisc_0.0.4, scales_1.2.1, GGally_2.1.2, ggforestplot_0.1.0, cowplot_1.1.1, ape_5.7-1, spaa_0.2.2, ggpubr_0.6.0, rioja_1.0-5, usedist_0.4.0, ANCOMBC_2.0.3, dplyr_1.1.2, qiime2R_0.99.6, ggplot2_3.4.2, tidyr_1.3.0, vegan_2.6-4, lattice_0.20-45, permute_0.9-7, stringr_1.5.0, phyloseq_1.42.0. The code used for metagenomics analysis is available at https://github.com/GaryZhangYue/Cecilio_2024_TC1_sand flies. The code used for modelling is fully disclosed in the supplementary materials file.

For manuscripts utilizing custom algorithms or software that are central to the research but not yet described in published literature, software must be made available to editors and reviewers. We strongly encourage code deposition in a community repository (e.g. GitHub). See the Nature Portfolio guidelines for submitting code & software for further information.

Data

Policy information about availability of data

All manuscripts must include a data availability statement. This statement should provide the following information, where applicable:

- Accession codes, unique identifiers, or web links for publicly available datasets
- A description of any restrictions on data availability
- For clinical datasets or third party data, please ensure that the statement adheres to our policy

The bacterial strain D. tsuruhatensis TC1 is available from the NCIMB using the accession number 43398 under a material transfer agreement with NCIMB. The metagenomics raw data generated in this study have been deposited in the NCBI GeneBank database, under the BioProject number: PRJNA1079352 (https://www.ncbi.nlm.nih.gov/bioproject/PRJNA1079352/con). All remaining data are available in this manuscript and its supplementary materials, as well as in the form of Source Data, provided with this paper.

Research involving human participants, their data, or biological material

Policy information about studies with human participants or human data. See also policy information about sex, gender (identity/presentation), and sexual orientation and race, ethnicity and racism.

Reporting on sex and gender	This study does not involve human participants, their data, or biologic materials.
Reporting on race, ethnicity, or other socially relevant groupings	This study does not involve human participants, their data, or biologic materials.
Population characteristics	This study does not involve human participants, their data, or biologic materials.
Recruitment	This study does not involve human participants, their data, or biologic materials.
Ethics oversight	This study does not involve human participants, their data, or biologic materials.

Note that full information on the approval of the study protocol must also be provided in the manuscript.

Field-specific reporting

Please select the one below that is the best fit for your research. If you are not sure, read the appropriate sections before making your selection.

- [x] Life sciences
- [ ] Behavioural & social sciences
- [ ] Ecological, evolutionary & environmental sciences

For a reference copy of the document with all sections, see nature.com/documents/nr-reporting-summary-flat.pdf

Life sciences study design

All studies must disclose on these points even when the disclosure is negative.

Sample size	No formal sample-size determination was performed. All experiments were designed based on our accumulated experience with sandfly-transmitted Leishmania parasites for more than 2 decades; samples size were chosen based on published studies and were based on biological variation within groups, to ensure reproducibility. The sample size (n) can be found in the figures and/or respective legends. Refs: Cecilio et al 2020; Dey et al 2018; Gomes at al 2012; Oliveira et al 2008; etc
Data exclusions	No data were excluded from the analyses.
Replication	We conducted at least two independent experiments per data panel. All results were reproduced in every independent experiment performed. Often times, statistical significance was attained in a single independent experiment.
Randomization	Sand flies were randomly assigned to the different groups. In this study sand flies of the same age were used, under the same conditions; therefore no covariates can impact the study results. When applicable, cages with BALB/c 6-8 week old female mice were randomly assigned to groups. Mice were equally divided into control and experimental groups.
Blinding	Whenever possible, the scientist responsible for doing the quantification of the data was blinded. Of note, in our context, blinding is not essential as experimental outcomes do not reflect subjective scoring.

Reporting for specific materials, systems and methods

We require information from authors about some types of materials, experimental systems and methods used in many studies. Here, indicate whether each material, system or method listed is relevant to your study. If you are not sure if a list item applies to your research, read the appropriate section before selecting a response.

Materials & experimental systems

n/a	Involved in the study
☒	☐ Antibodies
☒	☐ Eukaryotic cell lines
☒	☐ Palaeontology and archaeology
☐	☒ Animals and other organisms
☒	☐ Clinical data
☒	☐ Dual use research of concern
☒	☐ Plants

Methods

n/a	Involved in the study
☒	☐ ChIP-seq
☒	☐ Flow cytometry
☒	☐ MRI-based neuroimaging

Animals and other research organisms

Policy information about studies involving animals; ARRIVE guidelines recommended for reporting animal research, and Sex and Gender in Research

Laboratory animals	Six-week-old female BALB/c mice were obtained from Charles River laboratories and used in this study. Mice were housed under pathogen-free conditions at the NIAID Twinbrook animal facility (Rockville, MD) at 18-23°C room temperature, and 40-70% relative humidity, under 12h dark/light cycles, and with water and food ad libitum. Phlebotomus duboscqi sandflies were reared in house and used in this study (no older than 6 days after emerging).
Wild animals	No wild animals were used in this study.
Reporting on sex	Insect females are the vectors of Leishmania parasites. Only female mice were used.
Field-collected samples	No field-collected samples were used in this study.
Ethics oversight	All animal experimental procedures were reviewed and approved by the National Institute of Allergy and Infectious Diseases (NIAID) Animal Care and Use Committee under animal protocol LMVR4E. The NIAID DIR Animal Care and Use Program complies with the Guide for the Care and Use of Laboratory Animals and with the NIH Office of Animal Care and Use and Animal Research Advisory Committee guidelines. Detailed NIH Animal Research Guidelines can be accessed at https://oma1.od.nih.gov/manualchapters/intramural/3040-2/.

Note that full information on the approval of the study protocol must also be provided in the manuscript.

Plants

Seed stocks	N/A
Novel plant genotypes	N/A
Authentication	N/A
